# Are tinnitus burden and tinnitus exacerbation after cochlear implantation influenced by insertion technique, array dislocation, and intracochlear trauma?

**DOI:** 10.3389/fneur.2024.1477259

**Published:** 2024-10-30

**Authors:** F. Everad, R. L. Beck, A. Aschendorff, A. K. Rauch, Leonie Fries, S. Arndt, M. C. Ketterer

**Affiliations:** Department of Otorhinolaryngology, Faculty of Medicine, Medical Center – University of Freiburg, Freiburg, Germany

**Keywords:** cochlear implant, tinnitus, anatomy, psychometry, cochlea

## Abstract

**Introduction:**

Although numerous studies suggest that cochlear implantation (CI) generally alleviates the overall burden of tinnitus, certain patients experience tinnitus exacerbation following CI. The exact cause of this exacerbation is still uncertain. This prospective study aimed to investigate whether cochlear trauma, resulting from scalar dislocation of the electrode array, affected postoperative tinnitus intensity, tinnitus burden, and speech perception. Additionally, the influence of CI insertion technique, insertion depth, insertion angle, and cochlear morphology on postoperative tinnitus was assessed.

**Methods:**

We evaluated 66 CI recipients preoperatively at 2 days, 4 weeks, and 12- and 24-months following surgery. Digital volume tomography was employed to document scalar position, insertion depth, and cochlear morphology postoperatively. Speech perception was analyzed using Freiburg monosyllables. The tinnitus burden was evaluated using the tinnitus questionnaire, while the tinnitus intensity was quantified using a visual analog scale.

**Results:**

Study results pertaining to tinnitus intensity and burden did not reveal a significant difference in elevation regarding scalar position and dislocation after CI surgery compared to preoperative tinnitus levels. However, dislocation was only identified in four patients, and scala vestibuli insertions were observed in two patients. Comparing preoperative and 1-year postoperative outcomes, CI was noted to substantially reduce the tinnitus burden. When the speech processor was worn, the tinnitus intensity was significantly diminished. In comparison to round window (RW) insertion, the insertion technique cochleostomy (CS) did not exhibit a significant difference or a trend toward increased tinnitus intensity.

**Conclusion:**

This study demonstrates that CI significantly decreases the tinnitus burden. The observation implies that the electrical stimulation of the auditory pathway, facilitated by wearing the speech processor, significantly reduced the tinnitus intensity. The incidence of dislocations and scala vestibuli insertions has declined to the extent that it is no longer feasible to formulate statistically significant conclusions.

## 1 Introduction

Cochlear implant (CI) treatment is a well-established hearing rehabilitation option for patients with bilateral deafness, single-sided deafness (SSD), and asymmetric hearing loss (AHL) (1, 2). Furthermore, previous research has demonstrated that CI has a significant impact on reducing tinnitus burden and distress in all of the aforementioned indication groups (3–8). However, some patients have reported postoperative tinnitus exacerbation and distress following CI surgery, and the underlying causes are still uncertain.

Aschendorff et al. (9) had initially reported that scala tympani (ST) inserted electrode arrays lead to significantly better speech understanding compared to scala vestibuli inserted electrode arrays, which had been corroborated in larger study cohorts (10, 11). Additional studies (12, 13) have developed methods for measuring cochlear size three-dimensionally and assessing the cochlear length to prepare an electrode array with a more customized shape. Ketterer et al. (13) observed that electrode arrays tend to dislocate in smaller cochleae, especially in those with reduced height. In large study cohorts, James et al. (14) and Ketterer et al. (15) evaluated the impact of electrode array design and cochlear coverage. They reported that the angular insertion depth of the electrode array does not enhance speech discrimination. Nevertheless, the incidence of dislocations depends on the electrode array design. Speech discrimination is not affected by electrode array dislocation (15). To the best of our knowledge, no published study has evaluated the influence of scalar location and intracochlear trauma on tinnitus exacerbation and tinnitus burden. The aim of this prospective study is to evaluate whether cochlear trauma caused by electrode array dislocation influences tinnitus exacerbation and reduces the effectiveness of CI in alleviating tinnitus burden. Furthermore, our objective was to ascertain if cochleostomy (CS) affects tinnitus exacerbation or burden compared to the RW technique of the electrode array in a cochlear morphology-based study cohort.

## 2 Methods

### 2.1 Study and participants

**Figure 1:**
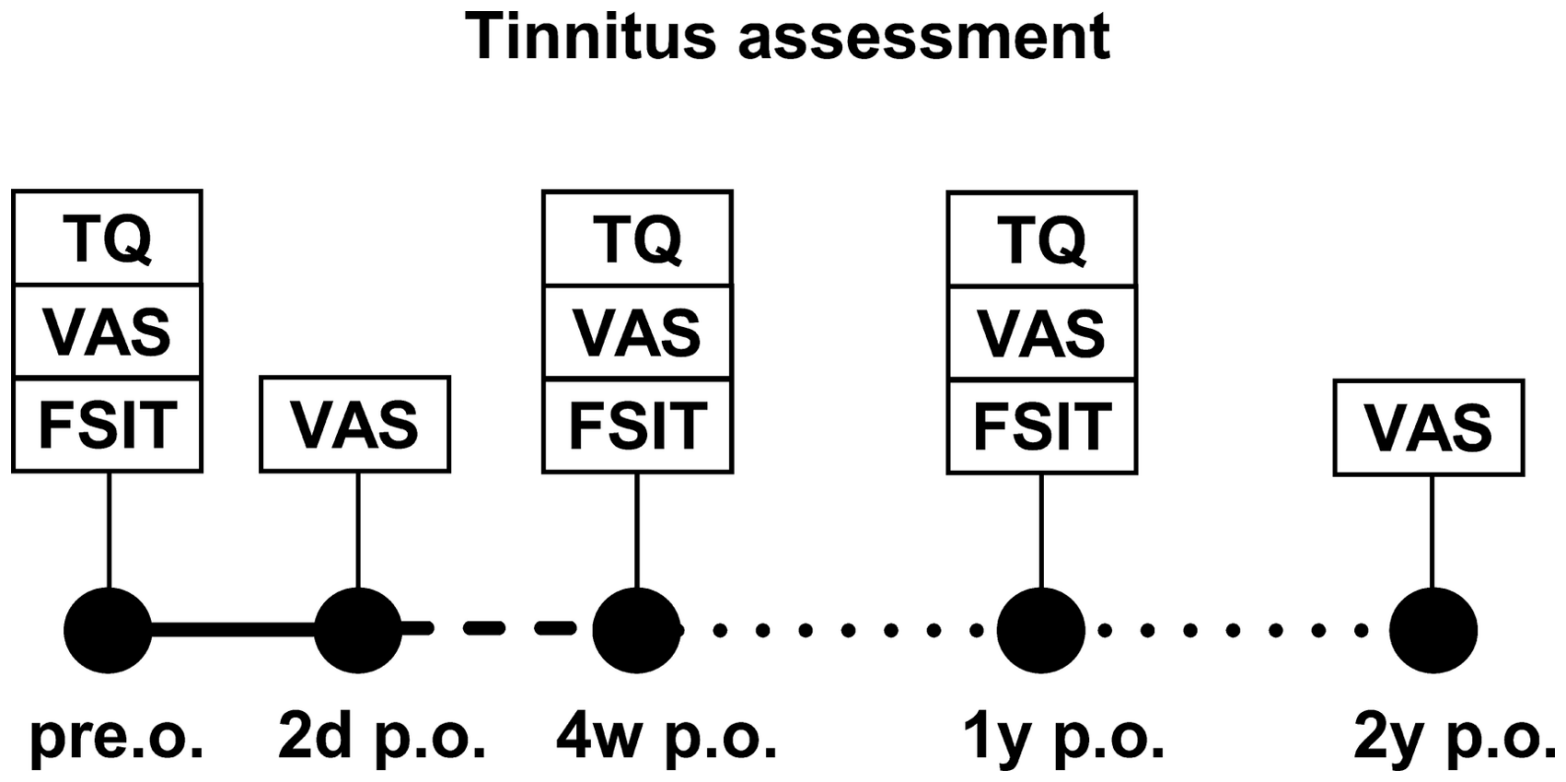
Study plan of tinnitus assessment at different time points after CI implantation: preoperative (pre.o.), 2 days postoperative (2d p.o.), 4 weeks postoperative (4w p.o.), 1 year postoperative (1y p.o.), and 2 years postoperative (2y p.o.). Assessment tools were the tinnitus questionnaire (TQ), visual analog scale (VAS), and aided and unaided Freiburg speech intelligibility test (FSIT).

This prospective study included adult patients who received CI between 2020 and 2022. The investigation encompassed all CI patients, regardless of whether they experienced preoperative tinnitus in the implanted ear. Contralateral tinnitus was not investigated. The study excluded patients with cochlear or vestibular malformations, hypoplasia, vestibular schwannoma, intellectual disabilities, or a history of post-labyrinthitis, stapedectomy, saccotomy, mastoidectomy, or reimplantation. There were no limitations in terms of implant type, manufacturer, or whether the patients experienced tinnitus preoperatively. Tinnitus distress was assessed on the day before implantation, 2 days postoperatively, after the initial fitting (approximately 4 weeks after implantation), and 1 and 2 years postoperatively (Figure 1). The German-version tinnitus questionnaire (TQ) (16) was employed to ascertain the tinnitus burden, whereas tinnitus intensity was assessed using the visual analog scale (VAS). In all TQ assessments, we did not differentiate between the speech processor being worn or not, because in some questions (e.g., regarding sleep quality), the requirement of wearing the speech processor could not be presupposed. Tinnitus intensity (VAS score) was generally measured without the speech processor. Conversely, at the 2-year follow-up, we requested that patients provide a description of the tinnitus intensity while wearing the speech processor and without it. Speech discrimination was measured using the Freiburg speech intelligibility test (FSIT) to establish a correlation between tinnitus and hearing outcomes (17).

### 2.2 Assessment tools for tinnitus distress and speech intelligibility

The TQ comprises 52 questions designed to collect information regarding the diverse aspects of tinnitus burden. It is organized into six subscales: (1) cognitive and (2) emotional distress; (3) intrusiveness; (4) auditory perceptual difficulties; (5) sleep disturbances; and (6) somatic complaints. Each question can be marked as “not true,” “sometimes true,” or “true,” with each response assigned a value of 0–2 points. Each sub-scale is assigned a score by adding up the points. A larger score indicates a more severe tinnitus burden.

The VAS is a simple instrument for evaluating tinnitus intensity. The horizontal line is a scale ranging from 0 (no tinnitus intensity) to 10 (maximum tinnitus intensity). Respondents were instructed to mark a point on the line corresponding to their current tinnitus intensity.

Speech discrimination is a recognized indicator of hearing progress after cochlea implantation and can be affected by tinnitus. Therefore, we incorporated the FSIT to ascertain whether there was a correlation between low speech perception and high tinnitus intensity. The speech perception threshold (determined by two-digit numbers) and the capacity to distinguish speech at suprathreshold levels (determined by monosyllabic nouns) are determined using the FSIT. In this study, speech discrimination was assessed employing monosyllabic nouns at 65 dB SPL with and without hearing aids preoperatively. After surgery, speech discrimination was assessed using monosyllables at 65 dB SPL with CI.

### 2.3 Radiological evaluation

A digital volume tomography (DVT) (New Tom 5G / GXL, Hillus Medical Engineering KG) was employed for the routine postoperative radiological evaluation of adult CI patients. The images were evaluated by three experienced head and neck surgeons, one of whom specialized in CI surgery. We analyzed the scans and measured the distances A and B as described by Escudé et al. (12) and the cochlear height as established by Ketterer et al. (13). Scalar dislocation, dislocation point, and insertion angle were evaluated using the methodologies outlined by Aschendorff, Ketterer, and Beck et al. (9, 13, 15, 18–20).

### 2.4 Statistical analysis and ethics committee

We conducted our statistical analysis using the Gnu R statistical computation and graphics system (GNU R, Version 3.6.2, Core Team, Vienna, Austria^1^), which was further enhanced with the packages NLME (Linear and Nonlinear Mixed Effects Models, Version 3.1, Pinheiro et al.^2^), ggplot2 (Version 3.3.1, Hadley Wickham^3^), and GraphPad Prism (Version 10, © 2023 GraphPad Software^4^). This prospective study was approved by the medical university’s Ethics Committee in accordance with the Declaration of Helsinki (Washington, 2002) (Number of Ethics Committee approval: 129/19, amendment: 240022) and registered in the German Clinical Trials Register (www.drks.de/DRKS, DRKS00034647).

## 3 Results

### 3.1 Study and participants

This study included 66 adult patients, of whom 10 reported no tinnitus preoperatively. At 2 days following surgery, 65 patients completed an initial postoperative assessment. One patient had to be excluded from further analysis due to the intraoperative diagnosis of an intracochlear schwannoma (exclusion criterion). The assessment was completed by 58 patients (88%) 4 weeks after implantation, 28 patients (42%) at 1 year, and 31 patients (47%) at the 2-year follow-up time point. We evaluated 38 left and 38 right ears from 36 female and 30 male patients. Furthermore, we included 28 patients with bilateral hearing loss, 25 patients with AHL, and 13 SSD patients. The descriptive statistics of the etiology, implant manufacturer, electrode array, and scalar position are presented in Tables 1, 2. Data concerning 55 RWs, 2 extended RWs, and 9 electrode arrays inserted via CS were incorporated and analyzed.

**Table 1:** Descriptive study cohort details evaluated preoperatively with study inclusion.

Etiology (*n*)	Progressive hearing loss: 22
	Sudden hearing loss: 17
	Unknown: 16
	Acute deafness: 2
	Otosclerosis: 2
	Trauma: 2
	Familial: 2
	Endolymphhydrops: 1
	Large vestibular aqueduct: 1
	Vestibular schwannoma: 1 (excluded)

One patient exhibited a vestibular schwannoma and was excluded from further analysis.

**Table 2:** Distribution table of (a) included manufacturer with electrode arrays and (b) scalar position.

(a)				
Manufacturer (*n*)	Cochlear™: 31	MED-EL: 22	Advanced bionics: 9	Oticon: 4
Electrode array (*n*)	CI612: 3 CI 622: 21 CI 632: 7	Flex26: 15 Flex28: 7	HiFocus™ SlimJ: 8 HiFocus™ Mid- Scala: 1	EVO®: 4

(b)				
Scalar position (*n*)	Scala tympani: 60	Scala vestibuli: 2	Tympani dislocated: 4	
Dislocation angle (°)			180°: 3 patients	360°: 1 patient

### 3.2 Overall postoperative tinnitus and speech discrimination

**Figure 2:**
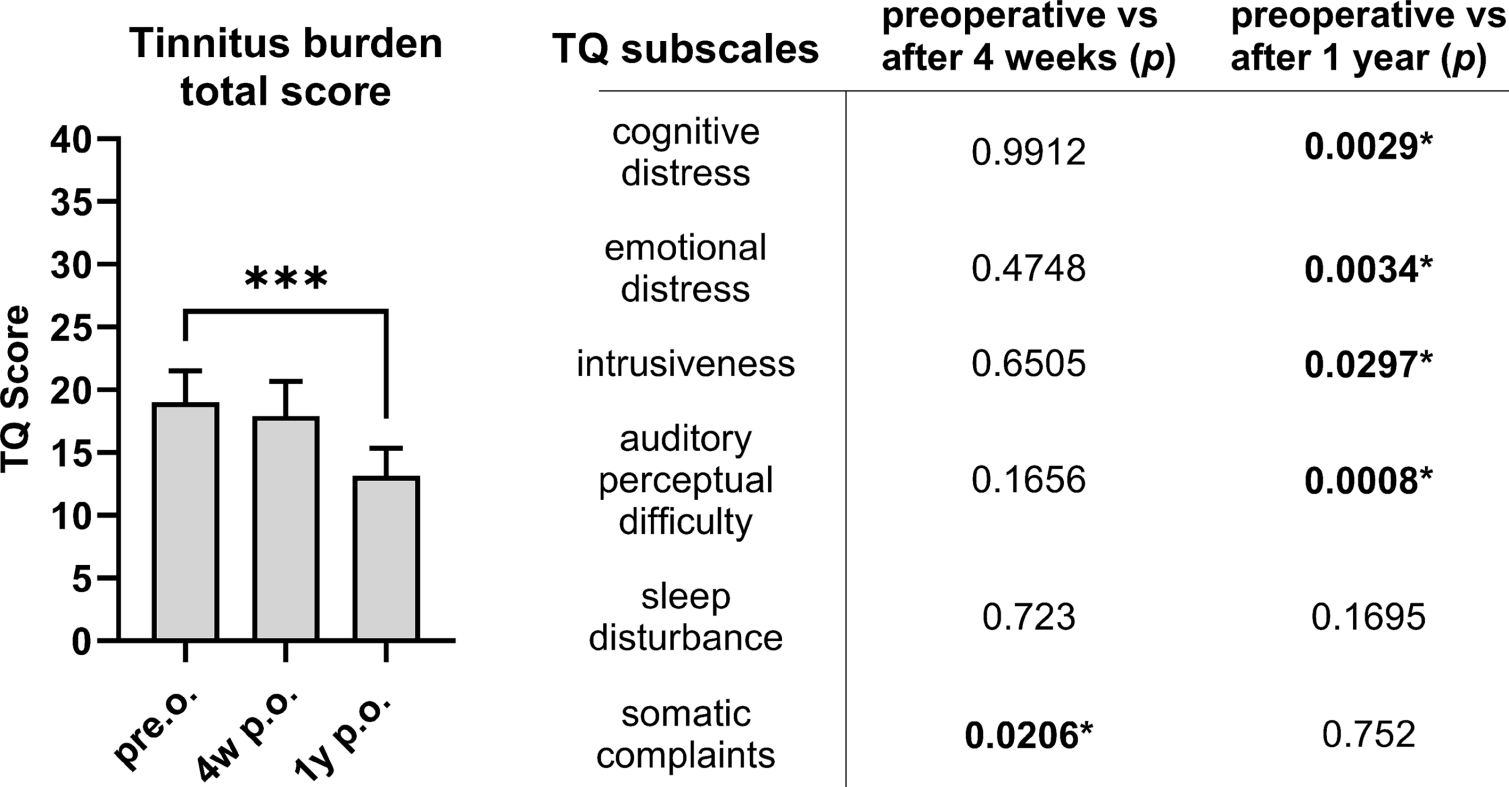
The tinnitus burden evaluated with the TQ total (left side) significantly decreased following CI comparing preoperative results and TQ total 1 year postoperatively. Differentiating the TQ subscales (right side), most aspects of tinnitus burden improve significantly 1 year postoperatively compared to preoperative. *p < 0.05 marked in bold. Sample size (*n*): TQ pre.o. *n* = 60; 4w p.o. *n* = 55; 1y p.o. *n* = 28.

**Figure 3:**
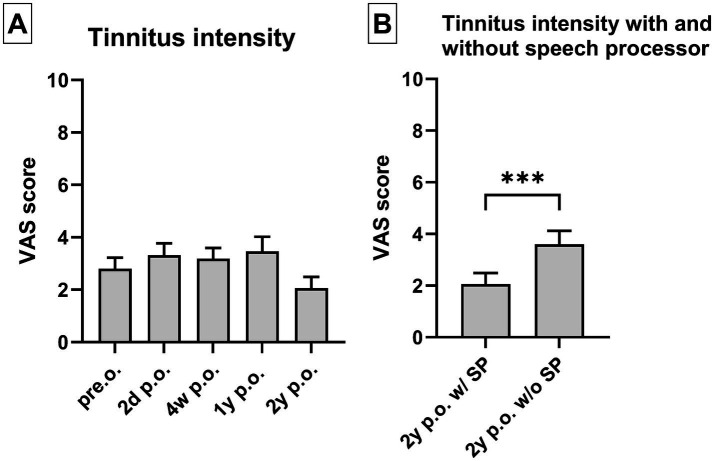
(A) The tinnitus intensity itself did not reduce significantly following CI surgery comparing preoperative results with the VAS evaluated 2 days, 4 weeks, 1 year, and 2 years postoperatively. (B) However, if patients wore the speech processor, tinnitus intensity was significantly reduced (2 years postoperative evaluation). *** *p* < 0.001. Sample size (*n*): VAS pre.o. *n* = 66; 2d p.o. *n* = 65, 4w p.o. 58; 1y p.o. *n* = 28; 2y p.o. *n* = 31.

The tinnitus burden of the entire study cohort decreased significantly (*p* = 0.0006) when the TQ total was compared to the results preoperatively and 1 year postoperatively (Figure 2). Compared to the preoperative levels, the TQ subscales 1 year postoperatively exhibited a substantial improvement in cognitive distress (*p* = 0.0029), emotional distress (*p* = 0.0034), intrusiveness (*p* = 0.0297), and auditory perceptual difficulties (*p* = 0.0008). However, the CI did not affect sleep disturbances (*p* = 0.1695) and somatic complaints increased compared to preoperative evaluation (*p* = 0.0206). Nevertheless, as depicted in Figure 3A, the tinnitus intensity assessed using the VAS remained consistent at 2 days, 4 weeks, and 1 and 2 years postoperatively. The tinnitus intensity assessed with the VAS was substantially reduced using the speech processor and electrical CI stimulation to stimulate the auditory system (Figure 3B). One patient, who did not have tinnitus prior to surgery, developed a temporary tinnitus postoperatively. Preoperatively, his VAS score was 0; however, it increased to 4 two days after implantation. However, his tinnitus intensity level had already dropped to zero at the 4-week assessment. Moreover, 5 patients demonstrated identical VAS scores before and after implantation.

When comparing preoperative measurements to those measured 4 days post-implantation, 8 out of the 56 patients with preoperative tinnitus complaints reported an increase in tinnitus intensity on the VAS. However, in all eight patients, this effect was transient, and their tinnitus intensity reverted to the initial preoperative level 12 months following CI.

Speech discrimination prior to implantation evaluated using the FSIT of monosyllables at 65 dB SPL was 1.3% ± 0.9 (without hearing aids) and 8.0% ± 2.3 (with hearing aids). It exhibited no correlation with preoperative tinnitus intensity or tinnitus burden. Significant improvements in FSIT were observed after 4 weeks (23.4% ± 3.3; *p* < 0001) and 1 year (53.3% ± 4.3, *p* < 0.0001) following implantation by CI.

### 3.3 Insertion techniques

**Figure 4:**
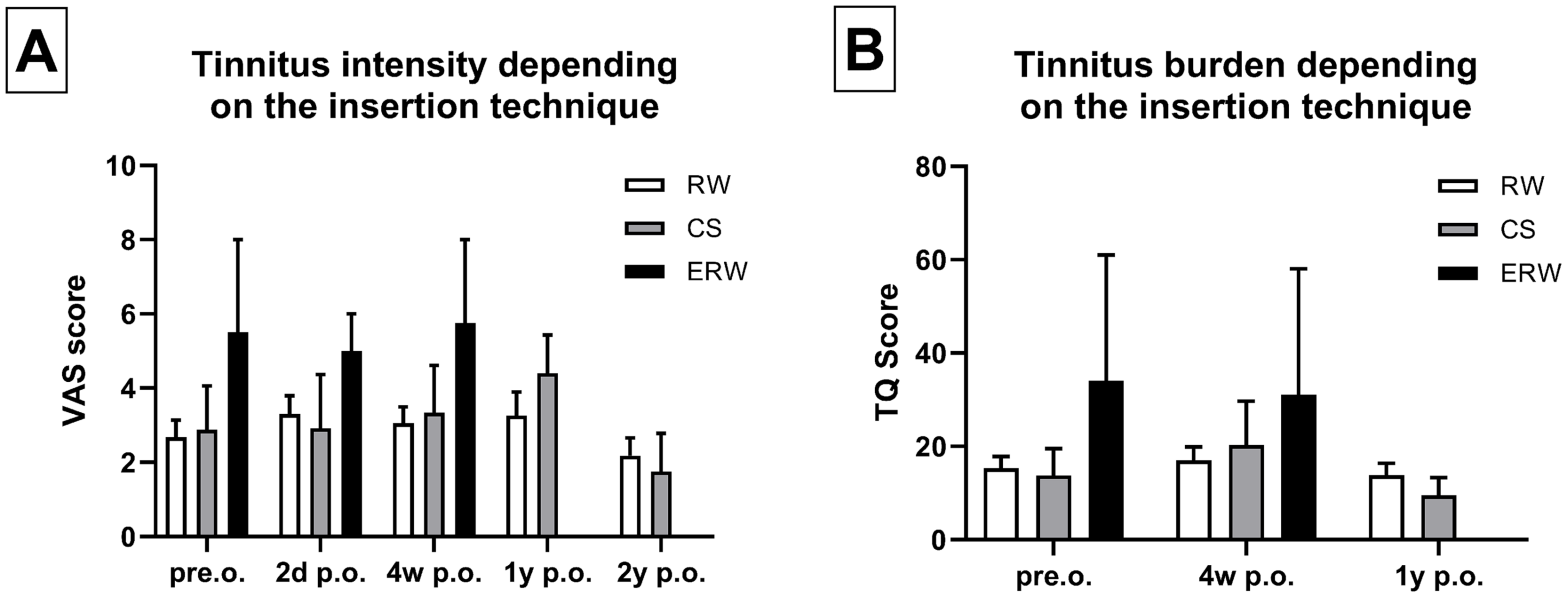
(A) Tinnitus intensity did not change significantly comparing the patients that underwent CS (=cochleostomy), RW (= round window) insertion, and ERW (=extended round window) insertion. (B) Similarly, the tinnitus burden did not change significantly comparing the patients who underwent CS, RW, and ERW. Sample size (*n*): RW pre.o. *n* = 48; 2d p.o. *n* = 44; 4w p.o. *n* = 23; 1y p.o. *n* = 28; 2y p.o. *n* = 28. CS pre.o. *n* = 8; 2d p.o. *n* = 6; 4w p.o. *n* = 6; 1y p.o. *n* = 5; 2y p.o. *n* = 4. ERW pre.o. *n* = 2; 2d p.o. *n* = 2; 4w p.o. *n* = 2; 1y p.o. *n* = 0; 2y p.o. *n* = 1.

Following surgery, we compared the tinnitus intensity evaluated with the VAS in relation to the insertion technique. We compared the electrode insertion technique using CS, round window (RW) insertion, and extended round window (ERW) insertion. Table 2 depicts that 55 patients underwent electrode insertion via RW, 9 via CS, and 2 patients underwent ERW-based electrode insertion. When assessed without wearing the speech processor, the tinnitus intensity did not change significantly based on the insertion techniques employed. Furthermore, we did not find any significant differences in tinnitus intensity comparing these three insertion techniques 2 days, 4 weeks, 1 year, and 2 years postoperatively (Figure 4A). These findings were consistent with postoperative tinnitus burden, as we did not note any significant changes depending on the insertion technique at 2 days, 4 weeks, and 1 year postoperatively (Figure 4B).

### 3.4 Electrode array dislocation

**Figure 5:**
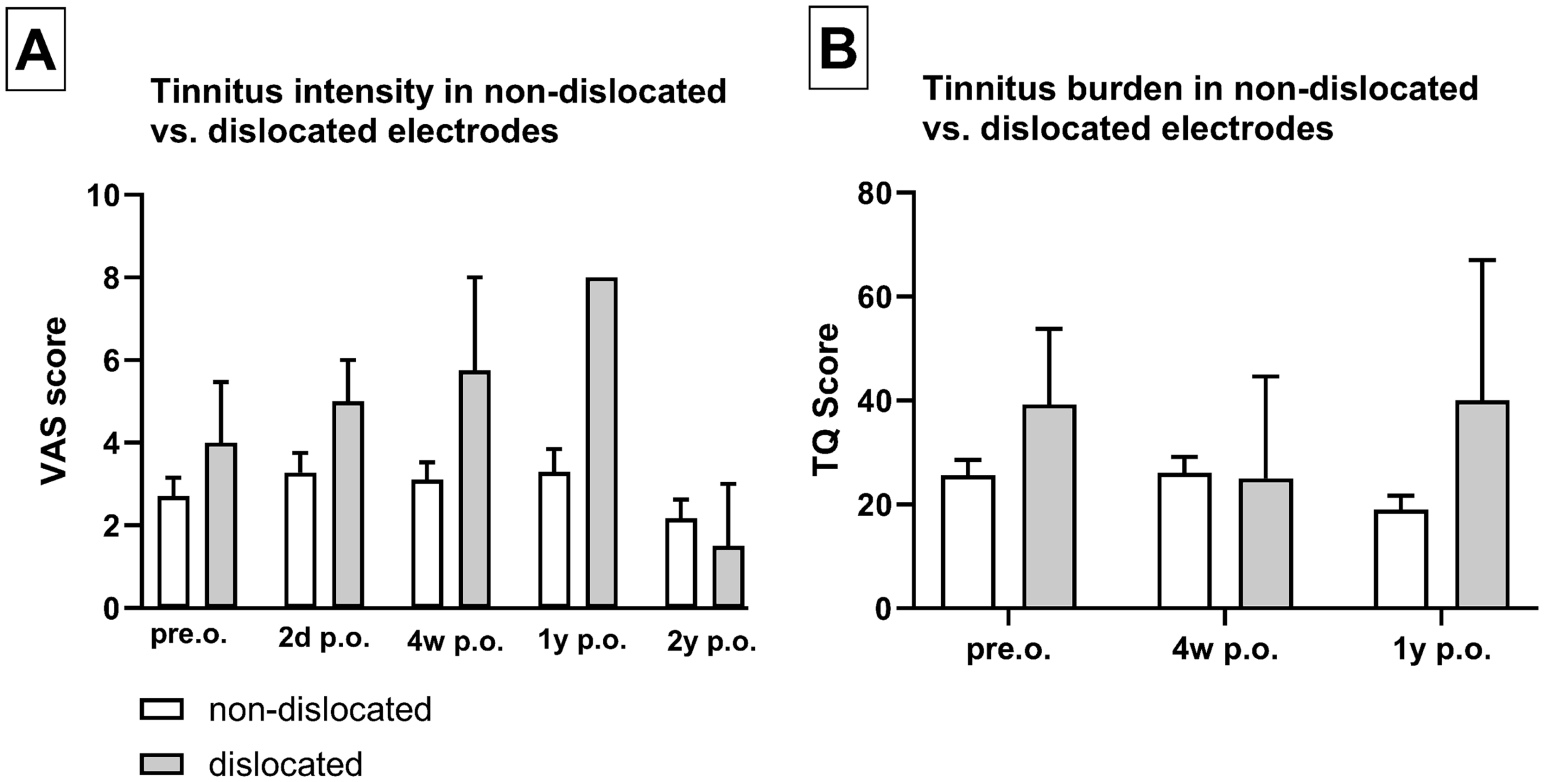
Dislocation of the electrode array did not show significantly increased tinnitus intensity (A) or tinnitus burden (B). Sample size (*n*): non-dislocated pre.o. *n* = 54; 2d p.o. *n* = 48; 4w p.o. *n* = 50; 1y p.o. *n* = 24; 2y p.o. *n* = 29. Dislocated pre.o. *n* = 4; 2d p.o. *n* = 3; 4w p.o. *n* = 3; 1y p.o. *n* = 1; 2y p.o. *n* = 3.

Table 1 illustrates the distribution of scalar electrode array positions. DVT reconstruction revealed that 60 patients received electrode insertion into the ST without electrode array dislocation. In total, 2 patients primarily underwent electrode insertion into the SV via CS, and 4 patients exhibited scalar dislocation from ST into SV. Three electrode arrays were dislocated at 180° (2 CA of Cochlear™ and 1 MS of Advanced Bionics) and one at 360° in the apical cochlear turn (Flex28 of MED-EL). The overall electrode array dislocation rate from ST was 6%, which corresponds to four patients. These patients did not demonstrate a significantly higher tinnitus intensity or tinnitus burden compared to patients who did not experience electrode array dislocation (Figure 5).

### 3.5 Cochlear morphology, insertion angle, and depth

**Figure 6:**
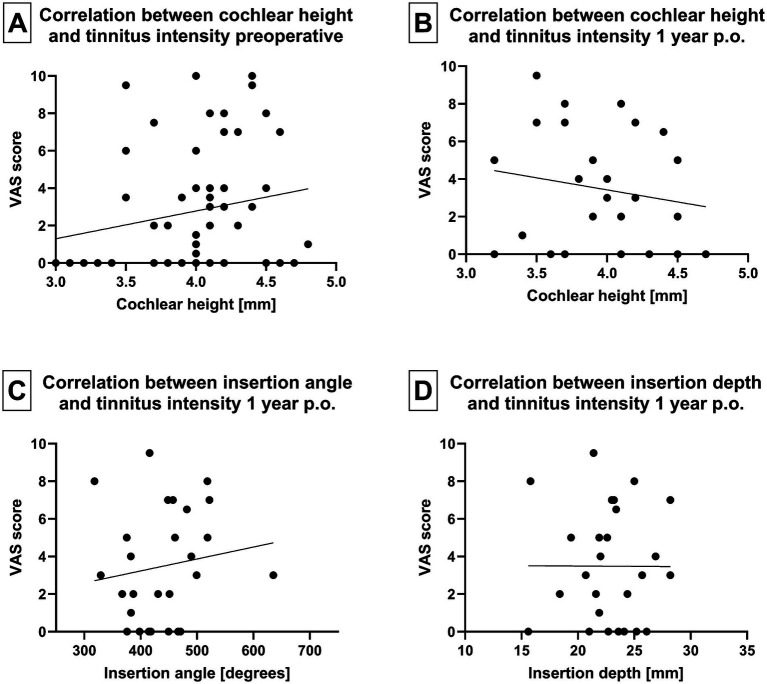
(A) Correlation between tinnitus intensity and cochlear height before implantation and (B) 1 year after implantation. (C) Correlation between array insertion angle and tinnitus intensity 1 year postoperatively. (D) Correlation between array insertion depth and tinnitus intensity 1 year postoperatively.

When analyzing cochlear morphology, neither cochlear height nor distance A or B exhibited any impact on preoperative tinnitus intensity (VAS score) or tinnitus burden (TQ total score). Similarly, there was no correlation between cochlear morphology and tinnitus intensity at 2 days, 4 weeks, 1 and 2 years postoperatively (Figures 6A,B). In addition, the tinnitus intensity was not influenced by the insertion depth or angle of the electrode array 1 year following implantation (Figures 6C,D).

## 4 Discussion

### 4.1 Cochlear implant and tinnitus

This study aimed to determine the impact of CI on both the intensity and burden of tinnitus. The evaluation of tinnitus burden employing the Tinnitus Questionnaire (TQtotal) revealed a significant reduction in tinnitus burden when comparing preoperative and 1-year postoperative results. These findings are consistent with the results obtained by Aschendorff et al. (21), Olze et al. (8), Bassouni et al. (22), Knopke et al. (4), Häußler et al. (5), and Ketterer et al. (6, 7). They observed that CI significantly alleviates the tinnitus burden following surgery across various indication groups, including in patients with SSD, AHL, or bilateral CI. Nevertheless, this study discovered that unaided tinnitus intensity was not significantly influenced by CI, as determined using a VAS. One potential explanation for this phenomenon is that electrode arrays are currently extremely thin and atraumatic, and the insertion and operation techniques are still as gentle and atraumatic as feasible. However, when participants wore the speech processor, there was a notable reduction in tinnitus intensity. This observation implies that the electrical stimulation of the auditory pathway, facilitated by the use of a speech processor, contributed to a significant decrease in tinnitus intensity. This effect was particularly evident when comparing tinnitus intensity 2 years postoperatively between individuals wearing the speech processor versus those who did not. In general, tinnitus was described by Olze et al. (8) as a symptom linked to the primary auditory cortex, often associated with cochlear deafferentation (23, 24) and accompanied by neuroplastic changes in the central auditory system. As previously described in multiple studies (8, 23, 25–27), electrical stimulation of the cochlea and the auditory system reduces the tinnitus burden in CI candidates. Although most patients experience a substantial reduction in their tinnitus burden because of CI, there are instances in which CI surgery is either causing tinnitus or negatively impacting the tinnitus intensity and burden (8, 28, 29). However, in our study, only 1 of the 10 patients (10%) developed new tinnitus, which was transient and resolved 4 weeks post-surgery. Nevertheless, 8 out of the 56 patients with preoperative tinnitus had a temporary increase in tinnitus intensity post-surgery, which subsided to the initial preoperative level of 12 months following CI. The causes of tinnitus persistence or exacerbation following CI remain unclear. One could hypothesize that the pressure wave generated by the insertion of the electrode carrier could induce auditory stimulation, particularly in patients with residual hearing, resulting in an increase in tinnitus. Conversely, tinnitus may be the subject of increased focus because of the general anesthesia and hospital stay. Additionally, the ear dressing that is applied or the hemotympanum that may occur following the CI could potentially isolate the ear, causing an increased focus on internal sounds. Therefore, the second aim of this study was to determine whether intracochlear trauma could be one of the potential causes for increased post-CI tinnitus.

### 4.2 Cochlear trauma and tinnitus

The primary objective of this study was to examine whether cochlear trauma resulting from electrode array scalar dislocation influences both the postoperative tinnitus intensity and burden. The patients included in this study demonstrated that intracochlear trauma due to electrode array dislocation does not appear to affect the risk for postoperative tinnitus exacerbation and/or tinnitus burden. Furthermore, tinnitus is not influenced by cochlear morphology and size. Multiple studies have previously addressed the impact of intracochlear trauma, e.g., on speech discrimination (11, 15) and the possibility of reimplantation (30, 31). Aschendorff et al. (9, 18) were the first to establish a connection between histological and radiological cochlear scans, suggesting that rotational tomography scans can effectively determine the scalar position of the electrode array. This was subsequently confirmed by Finley et al. (10) and Holden et al. (11). Electrode insertion into ST was found to be preferable due to its association with improved speech perception outcomes. Finley et al. (10) confirmed these findings in patients who received Advanced Bionics implants. In a related study, Aschendorff et al. (9) compared the perimodiolar electrode arrays of Cochlear™, specifically the Contour advance and the Contour electrode, and reported a scalar dislocation rate of 13.6% for the CA (Table 3). In recent years, manufacturers have also made significant efforts to create electrode arrays that are smaller and less traumatic. MED-EL, in particular, introduced the Flex26 electrode, which exhibited no signs of dislocation in our recently published investigation (19) and was characterized as a viable option for residual hearing preservation (32).

**Table 3:** Overview of own published studies from 2007 up to the present, detailing the investigated electrode arrays and their scalar positions.

Publication	Implanted in (year)	Investigated manufacturer (*n*)	Investigated electrode array (*n*)	Scalar position in %			
				ST	TD	SV	VD
Aschendorff et al. (9)	2000–2007	Cochlear (43)	Contour (22)	9.5	28.6	61.9	0
			CA (22)	72.7	13.6	13.6	0
Aschendorff et al. (33)	2000–2007	Cochlear (223)	Contour (21)	9.5	28.5	62	0
			CA (202)	65.3	18.8	14.4	1.5
Ketterer et al. (6)	2003–2010	Cochlear (403)	CA (368, s.f.a.)	68	19	11	2
Ketterer et al. (19)	2013–2019	MED EL (201)	F24 (28)	96.4	3.6	0	0
			F26 (15)	100	0	0	0
			F28 (139)	89.9	4.3	5.8	0
			F31.5 (19)	68.4	26.3	0	5.3
Ketterer et al. (15)	2013–2018	MED EL (168)	F24 (24)	95.8	0	4.2	0
			F28 (129)	92.2	3.9	3.9	0
			F31.5 (15)	73.3	20	0	6.7
		Cochlear (327)	CA (143)	72	15.4	10.5	2.1
			SMA (22)	90.9	0	9,1	0
			SSA (162)	97.5	0.6	1.9	0
Beck et al. (30)	2015–2020	Cochlear (81)	SMA (81)	93.8	2.5	3.7	0
This study		MED EL (22)	F26 (15)	100	0	0	0
			F28 (7)	71.4	14.3	14.3	0
		Cochlear (31)	CA (3)	33.3	66.7	0	0
			SMA (7)	100	0	0	0
			SSA (21)	100	0	0	0
		Advanced Bionics (9)	SlimJ (8)	87.5	0	12.5	0
			MS (1)	0	100	0	0
		Oticon (4)	EVO (4)	100	0	0	0

Dislocation rates have significantly decreased, and scala vestibuli insertions are significantly lower than in 2007. (s.f.a. = sufficient for analysis).

Table 3 presents an overview of the author’s published works from 2007 to the present regarding electrode arrays and scalar position. Dislocation rates have declined significantly, and scala vestibuli insertions are significantly lower than they were in 2007. Aschendorff reported that CI surgeons exhibit individual learning curves when it comes to the rate of scalar dislocations (33). Ketterer et al. (13) examined 403 ears implanted with a Contour Advance (Cochlear™) electrode, and after conducting a separate study (15), they demonstrated that ST dislocation rates are declining significantly (Table 3). We hypothesize that the atraumatic and small electrode array design observed in all the investigated electrodes as well as the enhanced surgical quality contributed to these results. As indicated by the results, RW should be preferred over CS, a large posterior tympanotomy should be conducted to enable adequate visibility, and the thinnest possible electrode arrays should be selected to minimize the risk of dislocation and scala vestibuli insertion. However, this study illustrates that tinnitus intensity and/or burden are not influenced or exacerbated by intracochlear trauma due to scalar dislocation, primary scala vestibuli insertion, altered angular insertion depth, or increased cochlear coverage.

## 5 Conclusion

In conclusion, this prospective study cohort confirmed that CI rehabilitation significantly mitigates the tinnitus burden. Tinnitus intensity diminishes considerably with the use of the speech processor and electrical stimulation of the cochlea and/or the auditory system. Cochlear trauma due to electrode array dislocation from the ST to the scala vestibuli, as well as primary scala vestibuli insertion and increased cochlear coverage, does not lead to a worsening of tinnitus burden or intensity. The utilization of RW insertions and the enhancement of electrode array design produced minimal dislocation rates and scala vestibuli insertions. Therefore, the dislocation rates currently are insufficient to perform a comprehensive statistical analysis.

## Data Availability

The raw data supporting the conclusions of this article will be made available by the authors, without undue reservation.

## Footnotes

^1^http://www.R-project.org

^2^https://CRAN.R-project.org/package=nlme

^3^https://ggplot2.tidyverse.org

^4^https://www.graphpad.com/
